# New and Emerging Approaches to Better Define Sleep Disruption and Its Consequences

**DOI:** 10.3389/fnins.2021.751730

**Published:** 2021-10-07

**Authors:** Bastien Lechat, Hannah Scott, Ganesh Naik, Kristy Hansen, Duc Phuc Nguyen, Andrew Vakulin, Peter Catcheside, Danny J. Eckert

**Affiliations:** Adelaide Institute for Sleep Health, Flinders University, Bedford Park, SA, Australia

**Keywords:** sleep disordered breathing, sleep apnea, insomnia, circadian rhythm, polysomnography, signal processing, apnea/hypopnea index, precision medicine

## Abstract

Current approaches to quantify and diagnose sleep disorders and circadian rhythm disruption are imprecise, laborious, and often do not relate well to key clinical and health outcomes. Newer emerging approaches that aim to overcome the practical and technical constraints of current sleep metrics have considerable potential to better explain sleep disorder pathophysiology and thus to more precisely align diagnostic, treatment and management approaches to underlying pathology. These include more fine-grained and continuous EEG signal feature detection and novel oxygenation metrics to better encapsulate hypoxia duration, frequency, and magnitude readily possible via more advanced data acquisition and scoring algorithm approaches. Recent technological advances may also soon facilitate simple assessment of circadian rhythm physiology at home to enable sleep disorder diagnostics even for “non-circadian rhythm” sleep disorders, such as chronic insomnia and sleep apnea, which in many cases also include a circadian disruption component. Bringing these novel approaches into the clinic and the home settings should be a priority for the field. Modern sleep tracking technology can also further facilitate the transition of sleep diagnostics from the laboratory to the home, where environmental factors such as noise and light could usefully inform clinical decision-making. The “endpoint” of these new and emerging assessments will be better targeted therapies that directly address underlying sleep disorder pathophysiology via an individualized, precision medicine approach. This review outlines the current state-of-the-art in sleep and circadian monitoring and diagnostics and covers several new and emerging approaches to better define sleep disruption and its consequences.

## Introduction

Sleep, along with diet and exercise, is essential for optimal health and wellbeing. However, globally, nearly 2 billion people are estimated to have one or both of the two most common clinical sleep disorders–sleep apnea (Benjafield et al., 2019) and insomnia (Roth et al., 2011). Most people with sleep disorders remain undiagnosed and untreated, and thus vulnerable to the major adverse health and safety consequences associated with untreated sleep disorders.

Current sleep apnea diagnostic approaches rely on traditional labor-intensive overnight sleep tests and subjective manual scoring approaches developed around the constraints of paper-based methods from the 1960’s. This approach, in combination with the advent of continuous positive airway pressure (CPAP) to reverse airway collapse during sleep (Sullivan et al., 1981), led to rapid advances in the modern field of sleep medicine. Although efficacious irrespective of underlying mechanisms, sub-optimal patient acceptance and use of CPAP remain problematic and warrant personalized treatments that better target underlying causal mechanisms. However, traditional sleep assessment methods fail to identify the specific underlying causes and consequences of sleep disorders for individual patients. For example, relationships between perceived sleep quality and/or sleepiness and objective sleep measures derived from traditional gold-standard polysomnography are either absent, weak, or inconsistent (Buysse et al., 2008; Sforza et al., 2015; Adams et al., 2016). In the case of insomnia, diagnosis relies on clinical evaluation since traditional objective sleep measures do not relate to disorder incidence, severity, or recovery. While the gold standard treatment, cognitive behavioral therapy for insomnia (CBT-I) is efficacious for many, it is ineffective or only partially effective for some patients (Trauer et al., 2015). This is potentially because, like CPAP for sleep apnea, CBT-I is a one-size-fits-all treatment regardless of the underlying causal mechanisms (Harvey and Tang, 2003). As such, usual care for sleep disorders typically relies on a trial-and-error treatment approach which often fails to identify the underlying causes of sleep disruption or adequately address patient symptoms and health consequences for which individuals seek treatment. Accordingly, this review focuses on highlighting new and emerging approaches to better define sleep and circadian disruption that underpins sleep disorders based on their underlying pathophysiology and accompanying health impacts.

## Current State of the Art for Sleep Recording

Current gold-standard methodology to quantify sleep relies on overnight polysomnographic (PSG) recordings. This includes collection of a wealth of neurophysiological data from electroencephalography (EEG), electrooculography (EOG), electromyography (EMG), electrocardiography (ECG), body position and movement, and respiratory-related signals including airflow, chest and abdominal motion, and oximetry. These signals are then manually reviewed and analyzed to classify wake, light through to deep non-rapid eye movement (NREM) (N1, N2, and N3), and rapid eye movement (REM) sleep in 30-s epochs. Transient cortical arousals (3–15 s) and longer awakening (>15 s) events are also manually scored on the basis of internationally standardized American Academy of Sleep Medicine (AASM) EEG criteria (Berry et al., 2017).

**FIGURE 1:**
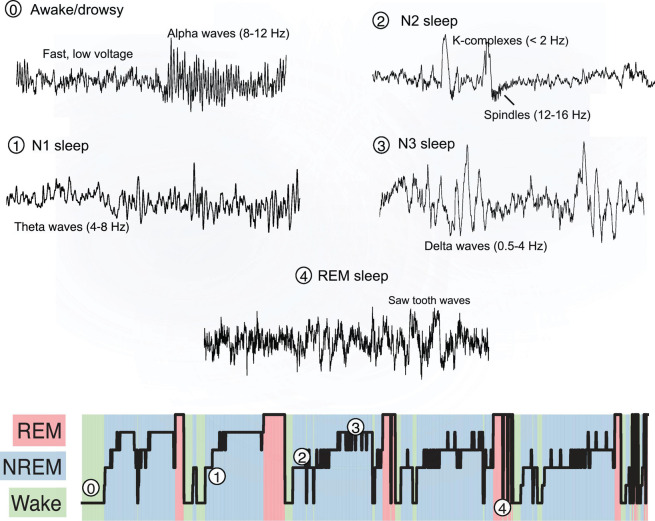
Electroencephalography activity during sleep.

Traditional polysomnography scoring evolved from observations of behavioral responsiveness changes coincident with changes in EEG patterns of activity at a time when chart recorders necessitated manual scoring, quite literally page-by-30-s-page (Rechtschaffen, 1968). This pattern-matching “bottom-up” approach to sleep medicine was based on the practical constraints with the technology available at the time, rather than being driven by an understanding of underlying sleep neurobiology. Although computerized systems have replaced paper-based recordings, and despite exponential advances in modern computing, sleep medicine remains predominantly based on these manual scoring methods from the 1960s. Manual scoring is labor intensive, and therefore costly, and captures only gross visually discernible EEG features with much poorer time and frequency resolution than is available within the data (Figure 1). Thus, EEG scoring into discrete 30-s epochs ignores that wake and sleep are continuous and dynamic states, whereby physiological features within epochs classified as wake can be present during sleep, and vice versa (Prerau et al., 2014; Scott et al., 2020). Manual scoring also has large intra- and inter-scorer variability, which remains problematic in sleep medicine despite AASM scoring criteria updates that attempt to reduce scoring variability (Ruehland et al., 2009; Magalang et al., 2013).

### A New Way of Thinking: Top-Down Sleep Signal Features Based on Underlying Neurobiology Rather Than Bottom-Up Measurement Convenience Guided Approaches

Automated sleep scoring methods using advanced signal processing and machine learning approaches to analyze polysomnography signals have been widely developed and can achieve good agreement against consensus-based traditional human scoring (Fiorillo et al., 2019). However, most of the focus has been on reproducing existing manual approaches (Tsinalis et al., 2016; Supratak et al., 2017; Chambon et al., 2018; Olesen et al., 2021). Thus, while these approaches are more standardized and time efficient, the fundamental limitations of traditional sleep metrics remain. Robust evidence to support causal relationships and clinical utility of most existing sleep metrics also remains sparse. Thus, the finer-grained quantifiable features within polysomnography data that may ultimately be more informative regarding underlying sleep mechanisms and quality continue to be largely ignored.

For example, EEG delta waves are tightly coupled in time and precede pulsatile changes in cerebral blood volume and cerebrospinal fluid flow during deep sleep (Fultz et al., 2019). Furthermore, a single night without sleep in healthy volunteers leads to β-amyloid accumulation (Shokri-Kojori et al., 2018). These findings support that delta waves during deep NREM sleep are a major driver of glymphatic clearance of metabolites from the central nervous system (Benveniste et al., 2020; Braun and Iliff, 2020). Wake/sleep transitions, such as potentially fatal microsleeps while driving, and a range of other physiological changes during sleep also occur on shorter timescales than assessed through traditional manual sleep scoring methods. For example, traditional scoring most likely misses potentially clinically informative neurophysiological features of synaptic downscaling, re-organization, memory and learning processes thought to occur during NREM and REM sleep (Tononi and Cirelli, 2006). Thus, conventional sleep scoring can only provide relatively superficial insights into brain activity and other physiological changes during sleep that are unlikely to be as sensitive or specific to underlying mechanisms as shorter-time scale features of sleep. Accordingly, a more physiologically guided, top-down measurement approach is clearly needed to provide greater neurobiological insight into sleep health and disease, and how sleep disturbance features relate to clinically relevant outcomes (Léger et al., 2018).

Defining evidence-based electrophysiological sleep markers is important in the age of precision medicine, particularly following rapid growth in minimally intrusive recording and consumer wearable devices that allow for sleep-related monitoring over prolonged periods in the home environment (Liu et al., 2017; Kim et al., 2019). These and other emerging technologies are likely to change many aspects of polysomnography, such as via printed electrodes (Norton et al., 2015) or tripolar concentric ring EEG (Besio et al., 2006), by helping to uncover aspects of sleep health not routinely measured. For example, markers of circadian misalignment are technically difficult to monitor, so remain notably absent from conventional sleep studies. Emerging evidence highlights the potential to estimate circadian phase using non-intrusive physiological data such as skin temperature, heart rate variability and activity (Suárez et al., 2020; Cheng et al., 2021). Blood pressure surges along with vasoconstriction and heart rate responses occur frequently during sleep, especially with swallowing (Burke et al., 2020), but are not currently routinely captured or assessed. Continuous measurement of a range of biomarkers such as cortisol secretion during sleep through skin sensor devices (Parlak et al., 2018) may also have clinical utility.

**FIGURE 2:**
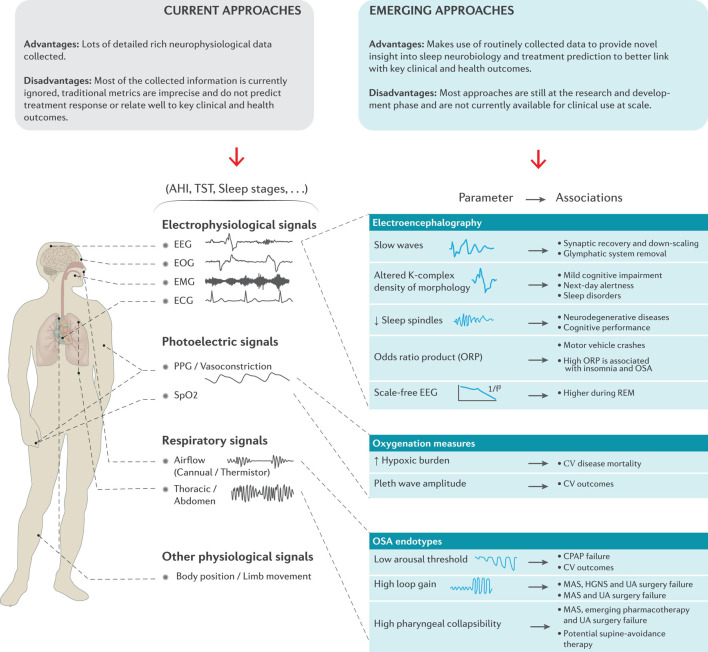
Schematic overview of the current metrics derived from standard polysomnography and the potential to make better use of these extensive neurophysiological signals provide novel insight into sleep neurobiology, treatment prediction and to better link with key clinical and health outcomes. Refer to the text for further detail. CPAP = continuous positive airway pressure, CV = cardiovascular, EEG = electroencephalography, EMG = electromyography, EOG = electrooculography, ECG = electrocardiography, HGNS = hypoglossal nerve stimulation, MAS = mandibular advancement splint, PPG = Photoplethysmography, REM = rapid eye movement, SpO_2_ = estimated arterial blood oxygen saturation and UA = upper airway.

Together, a range of new and emerging devices could routinely generate large volumes of sleep measurements over extended periods. This approach will require evidence to support clinical use and value, and software tools to assist clinicians to assess, analyze, and interpret sleep-omics (Redline and Purcell, 2021). To increase the uptake of new technologies in research and clinical settings, greater communication between sleep medicine experts and device manufacturers is needed. Rigorous standards for validation and evidence-based advances in medicine are required to ensure that new methods provide clinically useful insights that effectively and cost-effectively improve key patient outcomes (Depner et al., 2020). While not a complete list of all available approaches, the sections below highlight several examples of existing approaches and notable promising new and emerging methods based on underlying pathophysiology/neurobiology to move beyond key limitations of current sleep metrics. A schematic representation of some of these examples is provided in Figure 2.

## Key Components of the Polysomnographic

### Electroencephalography

This review focuses on novel sleep metrics derived from EEG collected clinically using routine polysomnography. Other reviews regarding potential neurobiological insights of sleep physiology and circadian rhythms through high density EEG and intra-cranial/depth EEG are available elsewhere (Mosqueiro et al., 2014; Saper and Fuller, 2017; Scammell et al., 2017).

#### Slow Waves

Slow waves (0.5–4.5 Hz) are the main feature of deep sleep and one of the fundamental electrophysiological features of synchronous neuronal “down states” of relative neuronal inactivity and “up states” as activity resumes (Nir et al., 2011). These waves are thought to play a major role in synaptic recovery and down-scaling to compensate for daily high neuronal activity and synaptic potentiation during wake (Tononi and Cirelli, 2006) and glymphatic system removal of metabolic waste products from the central nervous system (Benveniste et al., 2020; Braun and Iliff, 2020). Slow waves are ubiquitous during sleep, and decrease in quantity and magnitude with age (Chinoy et al., 2014). Several techniques have been developed to study specific aspects of slow waves, such as slow wave slope, absolute power, amplitude and phase in response to a range of experimental or naturalistic (e.g., aging) conditions (Massimini et al., 2004; Bersagliere and Achermann, 2010; Lazar et al., 2015; Lendner et al., 2020; Djonlagic et al., 2021). For example, the slope of half slow-waves (i.e., the slope between the up- and down-states) and slow wave amplitude/absolute power increase with sleep restriction and decrease with circadian phase, suggesting that sleep need and circadian rhythms have an effect on the shape and distribution of slow oscillations (Massimini et al., 2004; Bersagliere and Achermann, 2010; Lazar et al., 2015). Using the same features, reduced slow oscillations during sleep (low amplitude/absolute power) have recently been associated with poorer cognitive performance on a digit symbol coding test and the Trails B test in a large cross-sectional study of ∼3800 participants (Djonlagic et al., 2021). Many of these tools are available in open-source packages. With standardization, clinical validation and implementation, these novel metrics have substantial potential to provide unique insight into inter-individual vulnerability to specific health consequences in people with sleep disruption (Léger et al., 2018).

#### K-complexes

K-complexes are a form of isolated slow waves that provide unique insight into sleep stability and sleep disruption. They can occur spontaneously during sleep. However, K-complexes can also provide a sensitive marker of sensory disturbance to noise, respiratory and vibratory stimuli during sleep (Colrain, 2005; Scott et al., 2020; Lechat et al., 2021). Abnormal K-complex morphology (lower amplitude) and lower K-complex density (# per minutes) have been associated with the progression of amnestic mild cognitive impairment (pre-clinical phase of Alzheimer’s disease) in ∼70 patients (Liu et al., 2020). Abnormal K-complex morphology has also been associated with greater lapses in next-day alertness as measured using a psychomotor vigilance task (Parekh et al., 2019, 2021). At a population level, cross-sectional studies have suggested that a decrease in K-complex density may be a biomarker of sleep disorders, such as sleep apnea (Lechat et al., 2020). Further evidence regarding the functional significance of K-complexes is still emerging and warrants future investigation. This is likely to be facilitated via recent open-source tool developments (Parekh et al., 2015; Lechat et al., 2020).

#### Sleep Spindles

Sleep spindles are bursts of 11–15 Hz EEG activity and are another characteristic feature of NREM sleep that may provide a useful biomarker of sleep regulation and cognitive functioning (Diekelmann and Born, 2010; Djonlagic et al., 2021). Sleep spindles are influenced by genetics and vary widely across the lifespan and different demographics (Purcell et al., 2017). Higher spindle occurrence (and density) have been associated with better memory performance and vigilance (Lafortune et al., 2014; Hennies et al., 2016) in cross-sectional studies with moderate sample sizes (*n* < 100). In a clinical population of 47 patients with obstructive sleep apnea (OSA), greater sleep spindle activity was associated with better implicit learning (Stevens et al., 2021). A recent analysis of two large US-cohorts (n∼3800) also supported an association between higher spindle occurrence and spindle power with greater performance on multiple cognitive tests (Djonlagic et al., 2021). In addition, the coupling (proximity and phase differences) between slow oscillations and spindles was also predictive of cognitive performance, further supporting a role of spindles in memory formation (Hahn et al., 2020) and consolidation (Helfrich et al., 2019; Muehlroth et al., 2019). Together, these results may explain, at least in part, the association between abnormal spindle activity during sleep and neurodegenerative diseases such as Alzheimer’s disease (Gorgoni et al., 2016) and Parkinson’s diseases (Christensen et al., 2015). However, spindle detection is still a challenge and algorithm refinements on public benchmark datasets remain warranted (Warby et al., 2014; Lacourse et al., 2020). Furthermore, recent evidence suggests that the current definition of sleep spindles may be too restrictive and traditionally defined spindles may only be a small subset of a more generalized class of sigma oscillations during sleep (Dimitrov et al., 2021).

#### Fourier-Based Analysis of Sleep Signals: Quantitative Electroencephalography

**FIGURE 3:**
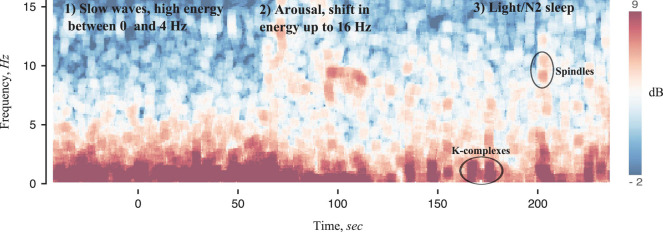
Spectrogram of sleep EEG signals using methods developed in Prerau et al. (2017). A transition from slow wave sleep (1) to N2 sleep (3) with an arousal in the middle (2) is observed. Slow wave sleep is characterized by high absolute power at frequencies less than 4 Hz and very little power at high frequencies, thus making identification of high frequency (8–16 Hz) arousals straight-forward. The transition from arousal to N2 sleep is also very specific, with a reduction in high frequency power, a sparse low frequency burst (likely reflecting K-complexes), sometimes followed by a burst of 12–16 Hz activity.

Sleep EEG is ideally suited to frequency and time-frequency analysis, since different stages or micro-elements (such as spindles, K-complexes, slow waves) have specific frequency characteristics (Steriade, 2006; Scammell et al., 2017), as shown in Figure 3. Power spectral analysis of EEG (sometimes referred to as quantitative EEG [qEEG]) provides a more sensitive and objective marker of neurophysiological features of sleep, some of which may be unique to specific patient phenotypes. For example, several studies have used qEEG to calculate the mean absolute power of given frequency bands (delta, alpha, theta, sigma, and beta), usually averaged over NREM and REM sleep, some of which have been shown to be predictive of insomnia (Krystal et al., 2002; Krystal and Edinger, 2010; Lunsford-Avery et al., 2021; Zhao et al., 2021) and OSA (D’Rozario et al., 2017; Appleton et al., 2019). Emerging evidence also suggests that qEEG markers are associated with vigilance and cognitive performance (Vakulin et al., 2016; Djonlagic et al., 2021; Mullins et al., 2021).

#### The Odds Ratio Product

The odds ratio product (ORP) is a novel EEG-derived metric that provides a continuous index of sleep depth and alertness (Younes et al., 2015; Younes and Hanly, 2016). ORP is calculated as a ratio of absolute power of different frequency bands over 3-s segments. The ratio ranges from 0 to 2.5, where 0 indicates very deep sleep and 2.5 is wide awake, and correlates well with the visual appearance of EEG across the night (Younes et al., 2015, 2020). ORP derived metrics may be useful for a wide range of clinical applications, such as phenotyping sleep disorders and associated health consequences (Younes and Giannouli, 2020; Azarbarzin et al., 2021; Younes et al., 2021). For example, sleep depth coherence between C3 and C4 channels measured using the ORP is associated with risk of motor vehicle crashes (Azarbarzin et al., 2021). A higher ORP during NREM sleep is also associated with the presence of OSA and insomnia, consistent with a more “alert” brain during NREM sleep in people with OSA and insomnia (Younes et al., 2021).

#### Scale-Free/Rapid Eye Movement Biomarkers

The scale-free component of neural activity (sometimes called “background brain activity” or “1/f” activity) is a further EEG component that may be an important biomarker of arousal level in human sleep (Lendner et al., 2020). Consistent with neuronal homeostatic and synaptic reorganization activity that takes place during REM sleep, 1/f activity is higher during REM sleep episodes. This observation may be especially important given the lack of targeted metrics designed to capture key physiological features of REM sleep. Eye movements, theta waves and atonia components require further investigation to test for relationships more comprehensively against other markers of REM sleep homeostasis and key clinical outcomes.

**FIGURE 4:**
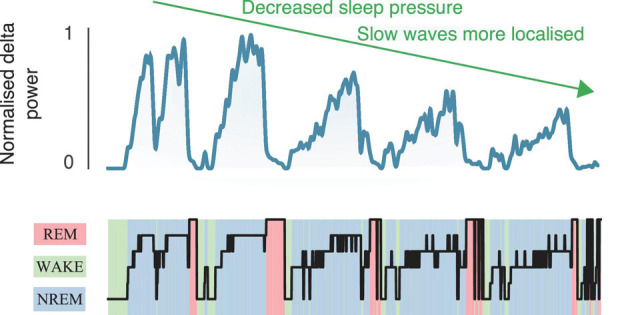
Cyclical variation in delta power across the night.

A limitation of all current biomarkers is the reliance on traditional manual scoring to express and evaluate summary values against conventional metrics with uncertain relationships with clinical endpoints. For example, absolute delta power, or ORP values, are usually averaged in NREM sleep. Spindles may be only detected in N2 sleep, and K-complex densities calculated in N2 and N3 sleep do not consider fluctuations in neurophysiological features across sleep cycles. EEG dynamics across sleep cycles are highly likely to be regulated by physiological processes such as circadian rhythms, brain metabolism, motor control learning, and memory consolidation processes (e.g., Figure 4). While averaging over traditionally scored sleep stages is convenient, it likely masks more subtle and potentially functionally important sleep-dependent changes over both short (<30 s) and longer cumulative time scales (minutes or hours). Secondly, current clinical utility of these biomarkers has mainly been studied cross-sectionally. Thus, well-designed randomized trials to investigate their potential additive benefit to sleep disorders management to improve health outcomes is warranted. Thirdly, methodologies used to calculate qEEG, ORP and other more fine-grained EEG elements are not standardized across research groups. Some methods are also not available under common license terms and therefore, independent cross-validation remains challenging.

### Oxygenation Measures

Pulse oximeters can continuously and minimally intrusively estimate blood hemoglobin oxygen saturation (SpO_2_) and are an almost ubiquitous device in the hospital environment (Jubran, 2004). Overnight pulse oximetry also provides a key requisite measure for the evaluation of sleep apnea (Netzer et al., 2001; Terrill, 2020). Standard traditional time-series measures derived from the oxygen saturation signal include mean and nadir overnight SpO_2_, time spent below SpO_2_ of 90% and the oxygen desaturation index (ODI), typically calculated as the number of 3 or 4% desaturations below baseline levels per hour of sleep. However, these metrics have their limitations and agreed standards for their calculation remain lacking. For example, the ODI is partly dependent on the criteria used to define SpO_2_ dip onsets, offsets, and duration. The ODI also only reflects the frequency of hypoxemic events and fails to reflect the degree and duration of hypoxemia and further oxidative stress through rapid reoxygenation (Punjabi et al., 2008). The physiological consequence of a 3 or 4% drop is also likely dependent on the baseline saturation level and temporal pattern of desaturation which can vary widely between individuals and comorbidities (Ayache and Strohl, 2018). Nonetheless, worse overnight hypoxemia derived from these traditional metrics has been associated with adverse health outcomes, such as increased blood pressure (Pengo et al., 2016; Su et al., 2021) and more recently atrophy of cortical and subcortical brain areas (Marchi et al., 2020). However, relationships with traditional hypoxia measures and important health/physiological outcomes are often weak, with inconsistent reproducibility between studies and cohorts (Pretto et al., 2014; Baumert et al., 2020; Linz et al., 2020; Terrill, 2020).

Other non-traditional parameters from SpO_2_ such as the delta index measures the mean absolute difference between successive points at constant time intervals (Levy et al., 1996; Magalang et al., 2003; Lin et al., 2009), saturation impairment index computed as the time integral over which SpO_2_ is below certain threshold levels (i.e., baseline, 90, 80, 70, 60, and 50% saturation) (Kirby et al., 1992), and the hypoxic burden index computed as the area under the time versus desaturation curve (SpO_2_ < 90%) divided by total sleep time (Azarbarzin et al., 2019; Baumert et al., 2020) have been derived and used in research settings. Some of these parameters have been associated with important health outcomes. For example, hypoxic burden measures that incorporate frequency, duration and magnitude of hypoxemia have recently been shown to predict cardiovascular disease mortality in different cohorts, whereas traditional PSG metrics such as the AHI and ODI do not (Azarbarzin et al., 2019; Baumert et al., 2020). Quantification of an easily measured index of sleep apnea-related hypoxemia has recently been used to predict incident heart failure (Azarbarzin et al., 2020). Accordingly, there remains considerable scope to better understand the precise mechanisms and characteristics by which hypoxemic and reoxygenation events during sleep contribute to cardiovascular and other end-organ damage, and to derive sensitive metrics to quantify these and other important health consequences. These recent findings highlight the potential for improvement beyond current traditional metrics. Through pulsatile changes in light absorption, oximeters can also provide potentially clinically useful markers of vasoconstriction responses during sleep (Catcheside et al., 2001; Jordan et al., 2003) that may be clinically useful predictors of cardiovascular risk (Hirotsu et al., 2020).

### Autonomic Signals

Assessment of autonomic nervous system activity during sleep is facilitated using photoplethysmography and ECG. The use of these signals in sleep medicine including new analytical methods and the potential insights they can provide has been covered in recent in-depth reviews (Fischer and Penzel, 2019; Ucak et al., 2021).

High increases in heart rate following apneic events are associated with 30–60% increases in mortality risk and non-fatal/fatal cardiovascular disease compared to normal heart rate responses (Azarbarzin et al., 2020). New evidence also suggests that heart rate variability during wakefulness could be a useful marker of OSA severity and excessive daytime sleepiness, whereby OSA severity is associated with reduced and less complex dynamics of heart rate variability (Qin et al., 2021). Pulse wave amplitude (a marker of vasoconstriction in the finger) features (e.g., amplitude, frequency) have been associated with hypertension, cardiovascular events and diabetes (Hirotsu et al., 2020). Similarly, a decrease in pulse arrival time (time delay of pulse propagation between two points such as heart and finger) as a result of apneic events, is a predictor of subclinical cardiovascular disease and future cardiovascular events (Kwon et al., 2021). Pulse wave amplitude and heart rate responses are also sensitive markers to sensory disturbances during sleep such as noise (Catcheside et al., 2002; Griefahn et al., 2008) and may therefore provide unique insights into downstream health effect of environmental sleep disturbances.

### Signal Coupling and Other Approaches

While an exhaustive list of sleep metrics is not the objective of this review, and recent detailed reviews are available elsewhere (Mendonça et al., 2019; Lim et al., 2020), a few key metrics warrant brief coverage.

Motor system disorders such as periodic limb movement (PLM) and REM sleep behavior disorders (RBD) are associated with adverse outcomes. For example, PLMs are associated with stroke and cardiovascular risk factors in certain patient populations (Lindner et al., 2012). RBD may be an early biomarker of subsequent synucleinopathies such as Parkinson’s disease (Claassen et al., 2010) and may increase the risk of stroke (Ma et al., 2017). RBD in people with Parkinson’s disease is also associated with faster motor progression and cognitive decline (Pagano et al., 2018). However, diagnosis of motor system disorders can be challenging. For example, screening questionnaires for RBD have variable sensitivity and specificity (Stiasny-Kolster et al., 2007; Li et al., 2010; Boeve et al., 2011). Thus, there is a need for better diagnostic approaches for motor system disorders. These include leg actigraphy for PLMs (Plante, 2014), more standardized quantifiable approaches using EMG signals during polysomnography (Frauscher et al., 2012) and novel 3D video analysis approaches (Waser et al., 2020).

The cyclic alternating pattern (CAP) is an additional sleep scoring system beyond traditional AASM sleep scoring which aims to quantify NREM discontinuity by characterizing phases of activation (A phases) and periods of inactivity (B phases) (Terzano et al., 2001). Automatic methods of CAP scoring have been proposed (Hartmann and Baumert, 2019) and have been applied to study and define NREM instability in large population-based studies (Buysse et al., 2010; Hartmann et al., 2020) and may provide unique insight into sleep neurobiology. CAP and its potential utility is discussed in detail in recent comprehensive reviews (Mendonça et al., 2019; Lim et al., 2020).

Several research groups have investigated the coupling between multiple physiological signals, such as heart rate with respiratory signals (named cardio-pulmonary coupling) (Thomas et al., 2005, 2018; Bartscha et al., 2012; Penzel et al., 2016). Coupling-based analyses have also been applied between sleep EEG and heart rate (Brandenberger et al., 2001). The theoretical concept of coupling-functions between different physiological systems has been recently generalized under the framework of network physiology (Bashan et al., 2012; Ivanov et al., 2016). A more in-depth review of these techniques and their potential to provide insight into sleep neurobiology and consequences of impaired coupling is available in the literature (Ivanov et al., 2016; Penzel et al., 2016; de Zambotti et al., 2018b).

## OSA Endotypes

**FIGURE 5:**
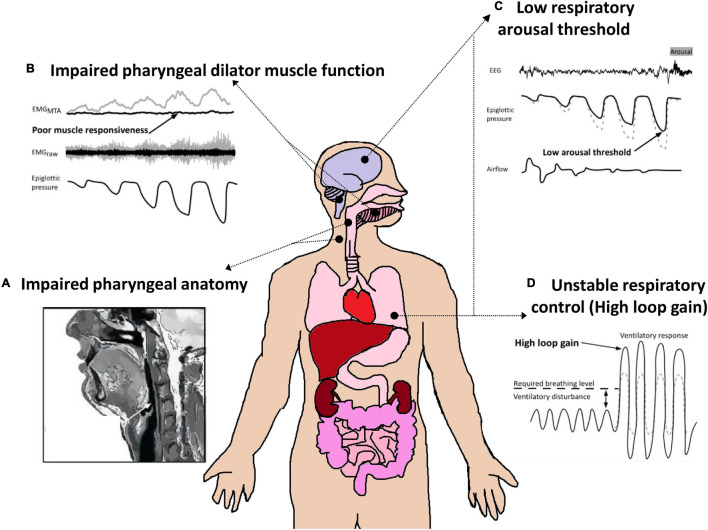
Schematic of the four key endotypic traits that contribute to OSA pathophysiology. **(A)** Impaired pharyngeal anatomy/collapsible upper airway. Non-anatomical endotypes include: **(B)** Poor pharyngeal dilator muscle function including poor responsiveness/activation to negative pharyngeal pressure/airway narrowing, **(C)** a low respiratory arousal threshold (waking up too easily to minor pharyngeal narrowing events); and **(D)** Unstable respiratory control/increased sensitivity to minor changes in CO_2_ (high loop gain). Each of these endotypes is a target for therapy or a “treatable trait.” Adapted from Carberry et al. (2018) and Aishah and Eckert (2019).

The underlying causes of the most common sleep-related breathing disorder, OSA, vary considerably between patients. Current evidence indicates that there are at least four key pathophysiological “phenotypes,” more recently termed “endotypes,” that contribute to OSA pathophysiology (Eckert et al., 2013; Eckert, 2018a; Malhotra et al., 2020). While impaired pharyngeal anatomy is the most influential endotype, the magnitude of impaired pharyngeal anatomy varies widely between patients. In addition, approximately 70% of patients also have one or more non-anatomical endotypes that contribute to their OSA (Eckert et al., 2013; Eckert, 2018a). These include impaired pharyngeal dilator muscle function during sleep, unstable control of breathing (high loop gain) and waking up too easily to minor airway narrowing events during sleep (low respiratory arousal threshold) (Figure 5). These advances in knowledge in OSA pathophysiology have major implications for targeted therapy through “precision medicine.” For example, detailed physiological studies in which the key OSA endotypes have been quantified and non-CPAP interventions delivered to improve one or more of the non-anatomical treatable traits can reduce OSA severity (Eckert et al., 2011; Edwards et al., 2012, 2016b; Sands et al., 2018a; Aishah and Eckert, 2019; Taranto-Montemurro et al., 2019; Op de Beeck et al., 2021). Identification of patients with a low respiratory arousal threshold endotype may be an important physiological predictor of CPAP treatment failure (Gray et al., 2017; Zinchuk et al., 2021) and the presence of a low arousal threshold endotype is associated with mortality (Butler et al., 2019). Similarly, identification of patients impairment in endotypes such as high loop gain and highly collapsible pharyngeal airways may be important predictors for non-CPAP treatment failure including upper airway surgery, mandibular advancement splint therapy, hypoglossal nerve stimulation, pharmacotherapy (Edwards et al., 2016a; Li et al., 2017; Aishah and Eckert, 2019; Op de Beeck et al., 2021) and potentially positional therapy (Eckert, 2018b).

However, current detailed physiological quantification of OSA endotypes is intrusive and far more complex and time-consuming to perform and analyze than standard polysomnography (Eckert, 2018a). Thus, this approach is impractical for clinical use. Accordingly, novel approaches to estimate the key OSA endotypes have been developed. These include more scalable advanced signal processing techniques (Sands et al., 2018a,b), machine learning approaches (Dutta et al., 2021) and algorithms (Edwards et al., 2014) which simply make better use of the existing rich neurophysiological and respiratory information acquired from diagnostic polysomnography recordings and standard clinical metrics such as age and BMI. Other strategies to estimate specific OSA endotypes include estimates based on a simple intervention during a CPAP titration study (Osman et al., 2020), the therapeutic CPAP level (Landry et al., 2017) and wakefulness upper airway physiology testing (Wang et al., 2018; Osman et al., 2019). These principles and recent proof-of-concept findings have opened multiple new lines of investigation for the development of more clinically feasible and scalable approaches to help better guide targeted therapy and precision medicine for OSA.

## Circadian Rhythms

### The Need to Assess Circadian Rhythms to Define Sleep Disruption

Aside from advances in PSG sleep and breathing metrics, new approaches are emerging in the assessment of circadian rhythms; another key determinant of sleep and its disorders (Borbély, 1982; Daan et al., 1984). These endogenous rhythms are ubiquitous, with nearly every cell in the human body influenced by a biological “clock.” The suprachiasmatic nucleus in the hypothalamus, colloquially termed the “master” clock, governs the timing of many circadian rhythms influential for sleep, including melatonin secretion, core body temperature (Cajochen et al., 2003), gene transcription and translation regulated clock behavior of nucleated cells throughout the body (Kondratova and Kondratov, 2012). Even non-nucleated red blood cells show circadian cycling of redox activity (O’Neill and Reddy, 2011). The effects of circadian rhythms on sleep disruption are most evident in circadian rhythm sleep disorders, such as delayed and advanced sleep-wake phase disorder, shift-work disorder, and non-24-h sleep disorder where the circadian phase (timing relative to clock time), amplitude of the rhythm, and/or period (duration of the circadian cycle) are poorly aligned with wake activities and environmental time cues, leading to disrupted sleep (Micic et al., 2016; James et al., 2017). Fortunately, disrupted circadian rhythms are treatable to improve sleep (Dodson and Zee, 2010).

Given the major role of circadian rhythms in mediating sleep patterns and behavior, methods to assess circadian rhythms across the different manifestations of sleep disruption are likely to be insightful. In chronic insomnia, circadian rhythm factors may importantly contribute to the underlying etiology and pathophysiology (Lack et al., 2008). Chronobiological interventions, such as bright light therapy, have been administered as a stand-alone treatment and combined with CBT-I to moderate effect (Jankù et al., 2020). Circadian rhythms could also play a role in OSA (von Allmen et al., 2018) and comorbid insomnia and OSA (COMISA) (Sweetman et al., 2021). Effects of circadian rhythms on respiratory control (Stephenson, 2003; Yamauchi et al., 2014) and hypoxia (von Allmen et al., 2018) have also been hypothesized and supported by recent evidence of circadian modulation of the key OSA endotypes (El-Chami et al., 2014, 2015; Puri et al., 2020). Circadian rhythms also have an influential effect on metabolism, diabetes, cardiovascular disorders, obesity, and the efficacy of a range of pharmacological interventions; factors often applicable to sleep disorder cohorts (Guo and Stein, 2003; Frazier and Chang, 2020; Ayyar and Sukumaran, 2021). Therefore, strategies to better define sleep disruption that incorporate circadian rhythm assessments have significant potential to improve diagnostic and targeted therapy outcomes.

### Current and Emerging Methods to Assess Circadian Rhythms

The current “gold standard” measure of circadian rhythms is salivary or blood dim-light melatonin onset (Arendt et al., 1985; Benloucif et al., 2008). This method involves measuring the concentration of melatonin (in pmol/mL) via a blood draw or via half-hourly saliva samples for at least 3–4 h before bedtime, under dim-light conditions (light intensity < 10 lux) while the individual remains relatively stationary and avoids consuming food and drinks (Sletten et al., 2018). Samples are processed and analyzed to estimate the clock time of melatonin rise onset (>10 pmol/mL), which is a marker of circadian phase. Another common measure of circadian rhythms in sleep research is core body temperature via an ingestible capsule or rectal thermistor. Frequent sampling of temperature across an extended period (>24 h), where conditions and activities that affect body temperature are controlled (e.g., air temperature, body movement, food consumption, and hot drink consumption), enables assessment of several aspects of the underlying core body temperature rhythm, including circadian phase, amplitude, and period. However, these assessments require carefully controlled laboratory conditions and access to specialized equipment generally infeasible for routine administration outside of circadian rhythm-focused sleep research studies. Fortunately, technologies and analytical methodologies are emerging that promise to facilitate simpler and improved assessments of circadian rhythms.

Emerging methods include advanced monitoring devices and biomathematical modeling to infer circadian rhythm metrics (Reid, 2019). Newer technologies include skin temperature sensors incorporated into consumer sleep trackers that detect the peripheral temperature rhythm to estimate circadian phase (Hasselberg et al., 2013). Electronic chips that can be implanted in body patches are also being developed to assess the cortisol rhythm via sweat (Upasham and Prasad, 2020), as well as other important clinical indicators such as the cortisol awakening response (Law and Clow, 2020). Rather than direct assessment of circadian rhythms, another approach is to infer circadian timing via the measurement of factors associated with circadian rhythms. Sleep timing data collected from wearable and non-wearable sleep trackers over an extended period are being incorporated into biomathematical models to infer circadian timing, since rest-activity rhythms are highly correlated with circadian timing (Cheng et al., 2021). Light sensors incorporated into newer wearable devices are also being used to infer circadian timing (Stone et al., 2020), since light is the strongest exogenous influencer (zeitgeber) of circadian rhythms. This information, potentially coupled with pupillometry assessment of an individual’s retinal responsiveness to light, enables inference of circadian timing, which may be useful for the diagnosis of circadian disruption in sleep disorders. More recent discoveries of genes with circadian oscillations (clock-controlled genes) raises the possibility that certain aspects of circadian rhythms may be amenable to assessment from blood samples (Cogswell et al., 2020). As these newer technologies mature, their implementation in clinical and research practice may result in new discoveries regarding the role of circadian rhythms in sleep disorders and their health-related consequences.

## Novel Measures of Environmental Factors That Can Affect Sleep

The sleeping environment affects sleep ability, but is minimally assessed in routine clinical practice. Consequently, sleep disruption may be misattributed to endogenous factors alone, ignoring the potential impact of exogenous factors. These include noise, light, temperature, and other factors that impact comfort within the sleep context. In a laboratory environment, these factors are typically well-controlled and designed to be conducive for sleep. However, as the assessment of sleep disruption shifts from the laboratory to the less well-controlled home environment, the assessment and consideration of environmental factors becomes increasingly important to understand mechanisms of sleep disruption.

Potentially the strongest exogenous influencer of sleep is noise, which can adversely affect sleep attainment and maintenance and fragment sleep to reduce total sleep time and quality (Muzet, 2007; Basner et al., 2014). The most common self-reported outcomes in response to road, rail and aircraft noise exposure are awakenings from sleep, increased sleep latency, and disruption to sleep continuity (Basner and McGuire, 2018). For example, patients in hospital intensive care units consistently rate noise as the most sleep disturbing factor (Freedman et al., 2001; Gabor et al., 2003; Elliott et al., 2013) and polysomnography results indicate poor and fragmented sleep, with a median of only 5 h sleep/24 h, only 3 min of uninterrupted light sleep and almost total abolition of deep and REM sleep (Elliott et al., 2014). However, to date, noise is rarely assessed as a potential sleep disturbing factor in either clinical or home setting contexts. Studies that have investigated the effects of noise on sleep quality have employed generalized metrics that focus on overall noise levels only and/or do not consider specific noise characteristics such as spectral content, time varying noise components, tonality and noise intermittency. These factors are important contributors to noise annoyance (Ioannidou et al., 2016; Schäffer et al., 2016; Oliva et al., 2017), are thus likely to contribute to sleep disturbance, and warrant assessment to better inform clinical decision-making.

## Scalable Approaches to Measure Sleep Including Multi-Night Assessments

There are two seemingly opposing challenges regarding sleep monitoring and diagnostics. There is a need for greater in-depth insight into the underlying neurobiology of sleep, yet there is also a need for less intrusive and user-friendly technology. Detailed, in-depth assessments and monitoring approaches as well as smarter use of existing signals and information derived from traditional polysomnography approaches are required to better understand sleep pathology. Yet, given the burden of disease and the scale of sleep disruption in the community, there is also a pressing need for less intrusive sleep tracking technology that can be readily and easily adopted in a home-based setting.

**FIGURE 6:**
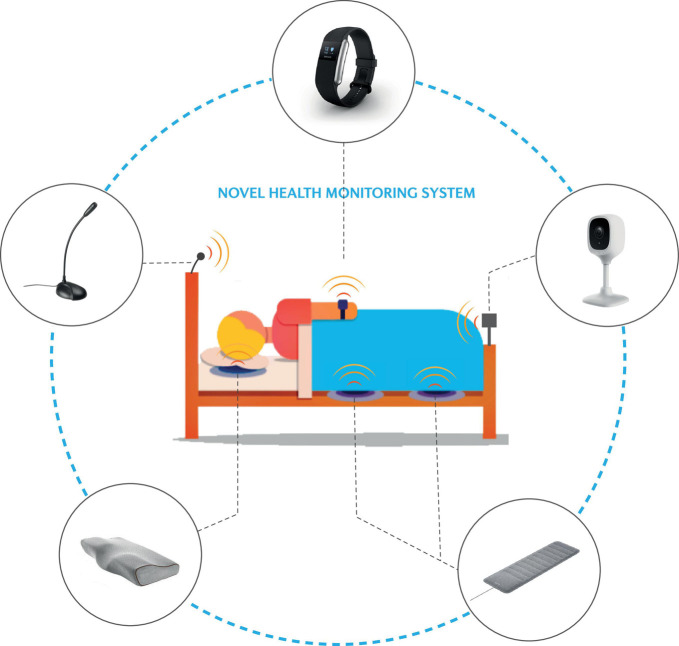
Schematic of novel and emerging approaches to monitor the sleeping environment and track key health measures via “the bedroom of the future.” Refer to the text for further detail.

A plethora of technologies have emerged to track sleep in the home setting (Figure 6). These include bedside Doppler (Zakrzewski et al., 2015; Tuominen et al., 2019) and instrumented mattresses for ballistographic assessment of heart rate, respiratory rate and body movements/position, which perform relatively well compared to polysomnography and are considerably easier to implement and use (Laurino et al., 2020). Similarly, wearable devices such as smart watches, rings, simplified EEG headbands, and actigraphy devices also provide similar performance in sleep/wake assessment (Griessenberger et al., 2013; de Zambotti et al., 2018a; Arnal et al., 2020; Chee et al., 2021; Scott et al., 2021). Infrared video has also been used to classify body motion to automatically score sleep and wake states (Wang et al., 2013), as well as monitor respiration, head posture, and body posture to detect abnormal breathing (Deng et al., 2018). Together, these devices open new pathways for non-invasive multi-night assessments in various sleep settings to support the clinical diagnosis and management of sleep disorders. This is especially important given that sleep disorder pathophysiology may show large variability between nights (Punjabi et al., 2020), and that variability and irregularity in some sleep components has been associated with downstream effects on health such as cardio-metabolic conditions (Linz et al., 2019a,b; Huang et al., 2020).

## Final Summary/Conclusion

New and emerging approaches to better define sleep and circadian disruption and its consequences offers considerable promise to move beyond the limitations of current sleep metrics and management. To improve outcomes, these approaches need to be underpinned by consideration for underlying neurobiology and will likely require a multisystem approach to capture the diverse impacts that sleep and circadian disruption can have on health and wellbeing. Development of practical, inexpensive methods to assess sleep and circadian disruption, its key contributors, and consequences at scale, including comprehensive, long-term remote monitoring has the potential to transform sleep medicine and management. This includes implementation of precision sleep medicine and targeted therapy approaches.

## Author Contributions

All authors contributed to drafting and/or revising one or more of the sections of this manuscript, provided feedback on the final version, and agreed to be accountable for the content of the work.

## Conflict of Interest

DE has a Collaborative Research Centre (CRC-P) Grant, a consortium grant between the Australian Government, Academia and Industry (Industry partner: Oventus Medical) and has research grants and serves as a consultant for Bayer and Apnimed. AV has received research grant funding and equipment from ResMed and Philips Respironics. PC has received research funding from Defence Science and Technology and received research grant funding and equipment from Philips Respironics. The funders Philips Respironics, ResMed, Oventus Medical, Bayer, Apnimed were not involved in the study design, collection, analysis, interpretation of data, the writing of this article or the decision to submit it for publication. The remaining authors declare that the research was conducted in the absence of any commercial or financial relationships that could be construed as a potential conflict of interest.

## Publisher’s Note

All claims expressed in this article are solely those of the authors and do not necessarily represent those of their affiliated organizations, or those of the publisher, the editors and the reviewers. Any product that may be evaluated in this article, or claim that may be made by its manufacturer, is not guaranteed or endorsed by the publisher.
