# Erratum: New and Emerging Approaches to Better Define Sleep Disruption and Its Consequences

**DOI:** 10.3389/fnins.2021.804589

**Published:** 2021-11-18

**Authors:** Frontiers Production Office

**Affiliations:** Frontiers Media SA, Lausanne, Switzerland

**Keywords:** sleep disordered breathing, sleep apnea, insomnia, circadian rhythm, polysomnography, signal processing, apnea/hypopnea index, precision medicine

Due to production errors, several corrections were omitted.

In “Key Components of The Polysomnographic,” “K-Complexes,” the sub-title should be “K-complexes.”

In “Key Components of The Polysomnographic,” “Sleep Spindles,” a sentence appears as follows: “In a clinical population of 47 patients with Obstructive sleep apnea (OSA) Headers are correct Confirmed, greater sleep spindle activity was associated with better implicit learning (Stevens et al., 2021).” This should instead read as follows: “In a clinical population of 47 patients with obstructive sleep apnea (OSA), greater sleep spindle activity was associated with better implicit learning (Stevens et al., 2021).”

In “Circadian Rhythms,” “The Need to Assess Circadian Rhythms' to Define Sleep Disruption,” a sentence appears as follows: “Chronobiological interventions, such as bright light therapy, have been administered as a stand-alone treatment and combined with CBT-I combined with CBT-I to moderate effect (Jankù et al., 2020).” This should instead read as follows: “Chronobiological interventions, such as bright light therapy, have been administered as a stand-alone treatment and combined with CBT-I to moderate effect (Jankù et al., 2020).”

The publisher apologizes for this mistake. The original version of this article has been updated.
